# Effects of aflatoxin B_1_ on growth performance, antioxidant status, immune response, and pro-inflammatory cytokine mRNA expression in ISA chicks

**DOI:** 10.3389/fvets.2022.993039

**Published:** 2022-09-13

**Authors:** Lele Hou, Huiling Qiu, Anping Li, Jihong Dong, Lianqin Zhu, Guowen Liu, Fu Chen

**Affiliations:** ^1^Institute of Nutrition Metabolic Disease and Poisoning Disease in Animals, Qingdao Agricultural University, Qingdao, China; ^2^Institute of Nutrition Metabolic Disease in Animals, Haidu College, Qingdao Agricultural, University, Laiyang, China; ^3^Key Laboratory of Zoonosis, Ministry of Education, College of Veterinary Medicine, Jilin University, Changchun, China

**Keywords:** aflatoxin B_1_, chick, growth, oxidant damage, pro-inflammatory cytokine

## Abstract

The research evaluated the effects of Aflatoxin B1 on growth performance, antioxidant status, immune response, and pro-inflammatory cytokine mRNA expression in ISA chicks. In total, 240 7-day-old ISA chicks were randomly assigned to four treatment groups. The control group comprised chicks fed a basal diet. The aflatoxin (AFB_1_)-treatment groups (T1, T2, and T3) comprised chicks fed the basal diet supplemented with AFB_1_ at concentrations of 5, 8, and 10 μg/kg, respectively. The growth performance, antioxidant status, immune responses, and pro-inflammatory cytokine mRNA expression in all groups were measured. In the T1 treatment group (receiving the lowest AFB_1_ dose), a reduction in the Newcastle disease virus antibody (NDV-Ab) titer, and increases in interleukin 2 (IL-2), IL-6, and interferon γ (IFN-γ) mRNA levels were observed on days 21 and 42 (*P* < 0.05). Treatment with the higher AFB_1_ doses (groups T2 and T3) reduced the chicks' growth performance on days 21 and 42, measured as reductions in body weight (BW) and average daily gain (ADG) compared with the control group. In the T2 and T3 groups, the total antioxidant capacity (T-AOC), glutathione peroxidase (GPX) and superoxide dismutase (SOD) activities, serum immunoglobulin A (IgA) and IgG levels, and IL-2, IL-6, and IFN-γ levels were also lower than in the control group. On days 21 and 42, these two groups also showed increased malondialdehyde (MDA) content, higher feed to gain ratio (F/G), and higher IL-2, IL-6, and IFN-γ mRNA levels than the control group (*P* < 0.05). The T2 and T3 groups also showed reduced T-AOC, NDV-Ab titer, IL-2 content, and GPx-1 mRNA levels on days 21 and 42 (*P* < 0.05), increased IL-6 and IFN-γ mRNA levels on day 21, and increased F/G and MDA content on day 42 (*P* < 0.05) compared with group (T1). Increased MDA content and IL-6 mRNA levels in the liver and ileum were observed in group T3 compared with group T2 on day 21, and lower IgM and IL-6 levels were observed on days 21 and 42 (*P* < 0.05). In conclusion, our data showed that AFB_1_ exposure resulted in dose-dependent oxidative and inflammatory damage, immunosuppression, and a decline in the growth performance of chicks.

## Introduction

Aflatoxins are toxic secondary metabolites produced by certain filamentous fungi, that occur widely in various foods and feeds (1, 2). Aflatoxin contamination can occur at every point along the food chain, from field to storage, including the feed-processing stage (3, 4). Aflatoxin B_1_ (AFB_1_) is the most toxic mycotoxin, with cytotoxic, genotoxic, and immunotoxic properties, and causes teratogenicity, mutagenesis and carcinogenesis (5, 6). AFB_1_ contamination can also cause the destruction of nutrients in raw materials or feed, reduce the palatability and nutritional value of feed, cause acute and chronic poisoning in animals, and lead to acute death (6, 7) and then cause significant economic losses.

Many studies have reported that poultry production is susceptible to AFB_1_ and that AFB_1_ contamination in feedstuffs poses a considerable threat to the growth and health of broiler chickens including weight gain, feed intake, and feed conversion ratio (8–11), and the growth and egg production of laying hens including egg production, egg size, and egg quality (12, 13) through toxic of the liver, kidneys, gastrointestinal tract and immune system. The immunosuppressive effect produced by aflatoxin AFB1 can be directly reduced effectiveness of vaccination programs, increased risk of infectious diseases, and high mortality (9).

However, most of the studies about negative effect of AFB1 were carried out on broilers and adult layers, the present study evaluated the toxic effects of AFB_1_ on growth performance, antioxidant status, immune response, and pro-inflammatory cytokine mRNA expression of pre-42-day-old ISA Chicks in order to improve the further understanding of AFB_1_ on the growth, health and immune suppression on egg production early of laying chicks, and provide theoretical basis for safe and healthy breeding of laying hens.

## Materials and methods

### Animals and treatment

In total, 240 1-day-old male chicks (ISA) were purchased from Institute of Animal Husbandry and Veterinary Medicine of Shandong Academy of Agricultural Sciences on day 0 after-hatching and randomly assigned to environmentally controlled brooder cages. Before the start of the experiment, all chicks were fed a basal diet for 7 days. The basal diet was a corn soybean- meal-based diet (Table 1), formulated to meet the nutritional requirements of ISA chicks aged 1–60 days. On day 7, the chicks were AFB_1_ assigned to four experimental treatment groups, each with six replicate pens, containing 10 chicks. The AFB_1_ content in the basal diet was confirmed with high-performance liquid chromatography tandem mass spectrometry (HPLC-MS; 1290 Infinity, Agilent, USA). The AFB_1_ content in the basal diet was 0, 5, 8, and 10 μg/kg according to the EU Commission Recommendation (14) and the Chinese Hygienic Standard for Feeds (15) guideline for the feed of young birds. The control group was fed the basal diet without AFB_1_ supplementation. The three AFB_1_ treatment groups, T1, T2 and T3, were fed the basal diet supplemented with AFB_1_ at concentrations of 2.70, 5.70 and 7.70 μg/kg, respectively. Based on the HPLC-MS analysis, the final AFB_1_ concentrations in the control, T1, T2, and T3 treatment groups feed were 2.30, 5.29, 8.43, and 10.90 μg/kg, respectively.

**Table 1:** Formulation and proximate composition of the basal diet.

**Composition**	**Content** **(%)**	**Nutrient**	**Content** **(%)**
Maize	64.0	Gross energy (MJ/kg)	11.9
Soybean meal	28	Crude protein	19.40
Fish meal	2.0	Calcium	1.00
Duck oil	1.9	Available phosphorus	0.43
Dicalcium phosphate	1.50	Lysine	1.00
Limestone	1.00	Methionine	0.51
Salt-NaCl	0.30	Methionine + cystine	0.83
DL-Methionine	0.20	Tryptophan	0.22
Choline chloride	0.10		
Mineral premix^†^	0.50		
Vitamin premix^‡^	0.50		
Total	100		

† The mineral premix supplied the following per kilogram of complete feed: CuSO_4_, 6 mg; ZnSO_4_, 80 mg; FeSO_4_, 80 mg; MnSO_4_, 100 mg; KI, 0.35 mg; CoCl_2_, 0.4 mg.

‡ The vitamin premix supplied the following per kilogram of complete feed: vitamin A: 4,000 IU; vitamin D_3_: 800 IU; vitamin E: 10 IU; vitamin K: 0.5 mg; vitamin B_2_: 3.6 mg; vitamin B_1_: 1.8 mg; vitamin B_12_: 0.01 mg; folacin: 0.55 mg/kg; pantothenic acid: 10 mg; niacin: 30 mg; biotin: 0.15 mg; choline: 1,300 mg.

All animal experiments were approved by the Qingdao Agricultural University Animal Care and Use Committee (Qingdao, China) in accordance with Laboratory Animal-Guidelines for the Ethical Review of Animal Welfare (GB/T35892-2018, National Standards of the People's Republic of China). During the experiment, the chicks were housed in a closed and ventilated building under continuous light. The room temperature was maintained at 32–34°C for the first 3 days, and then gradually reduced by 3°C/week until a temperature of 24°C was reached. The room was maintained at 24°C for the remainder of the experiment. Over the entire experimental period of 42 days, water and feed were provided *ad libitum*. All the chicks were inoculated with the Newcastle disease virus (NDV) vaccine (La Sota strain) on day 7, the attenuated infectious bursal disease virus (IBDV) vaccine on day 12, NDV on day 21, and IBDV on day 27.

### Growth performance

On day 42, body-weight (BW) and feed consumption (FC) were recorded, and the average daily gain (ADG) and feed conversion ratio (FCR) were calculated with the following formulaes:


$$\begin{array}{l} \text{ADG }(\text{g})\;=\;[\text{final weight }(\text{g})\;-\text{ initial weight }(\text{g})]\\ \text{ / feeding days }(\text{d})\\ \text{FCR }(\%)\;=\;[\text{total feed consumption }(\text{g})\;/\\ \text{ total final weight }(\text{g})\;-\text{ total initial weight }(\text{g})]\\ \;\times \text{ 100}\%. \end{array}$$

### Serum Sampling and analysis

Following the euthanization of the chicks on day 21 or 42, blood samples were drawn from the hearts of five randomly selected chicks in each treatment group. The blood samples were centrifuged at 3,000 × *g* for 15 min, and the serum was collected and stored at −20°C for further analysis. The glutathione peroxidase (GPX) activity, superoxide dismutase (SOD) activity, catalase (CAT) activity, total antioxidant capacity (T-AOC), and malondialdehyde (MDA) content in the sera were determined with assay kits (Nanjing Jiancheng Bioengineering Institute, Nanjing, China), according to the manufacturer's instructions. Serum immunoglobulin G (IgG), IgM, IgA, IL-2, IL-6, IFN-γ, anti-NDV antibody (NDV-Ab) titer, and anti-IBDV antibody (IBDV-Ab) titer were analyzed with enzyme-linked immunosorbent assay (ELISA) kits (Lair Biotechnology Co., Ltd., Hefei, China) according to the manufacturer's instructions.

### Tissue sampling and analysis

Following the euthanization of chicks on day 21 or 42, the liver, glandular stomach, ileum and spleen were collected from three randomly selected chicks and immediately processed in liquid nitrogen before further analysis of the mRNA expression of IL-2, IL-6, IFN-γ, and cellular GPX (GPx-1). Briefly, the total RNA was extracted from the tissues with TRIZOL Reagent (Sangon Biotech Shanghai Co., Ltd., China). The RNA was then reverse transcribed in 40 μl of reaction mixture according to the manufacturer's instructions (Sangon Biotech Shanghai Co., Ltd., China) and stored in liquid nitrogen. The Primer Premier 5.0 software (PREMIER Biosoft International, USA) was used to design specific PCR primers (Table 2).

**Table 2:** Primers used for quantitative real-time PCR analysis.

**Target genes**	**Sequences of nucleotide (5^′^−3^′^)**	**Fragment length (bp)**	**GenBank accession no**.
*β-actin*	agt gtc ttt ttg tat ctt ccg cc	147	NM_205518.1
	cca cat act ggc act tta ctc cta		
*GPx-1*	tct ac ctg gta act ttc gag caa	147	NM_001277853.2
	cct tta ttg cag agc ctc ctt		
*IL-2*	gaacctcaagagtcttacgggtcta	111	AF000631.1
	acaaagttggtcagttcatggaga		
*IL-6*	aaatccctcctcgccaatct	106	NM_204628.1
	ccctcacggtcttctccataaa		
*IFN-γ*	aagtcatagcggcacatcaaac	153	NM_205149.1
	ctggaatctcatgtcgttcatcg		

GPx-1, cellular glutathione peroxidase; IL-2, interleukin 2; IL-6, interleukin 6; IFN-γ, interferon γ.

Quantitative real-time polymerase chain reaction (qPCR) was performed with SYBR^®^ Premix Ex Taq™ II (Sangon Biotech Shanghai Co., Ltd., China) on the Applied Biosystems^®^ 7500 Fast Real-time PCR System (Applied Biosystems, Foster, USA) according to the manufacturer's instructions. The experiment was repeated in triplicate. The ratios of mRNA levels to that of the β-actin mRNA internal control were used to statistically compare the different treatments by 2^−Δ*ΔCt*^ method.

### Statistical analysis

All data are expressed as means ± standard deviations (SD), and were analyzed with one-way ANOVA to compare the means and with a multivariate general linear model (GLM) procedure in IBM SPSS for Windows version 22.0 (SPSS, Chicago, USA). The least significant difference (LSD) and Dunnett's T3 test were used to evaluate the differences between means. “Pen” was defined as the experimental unit for statistical purposes, and all calculations were generated based on pen averages. Differences were considered statistically significant at *P* < 0.05 for all tests.

## Results

### Exposure to AFB_1_ dose-dependently reduced growth performance

**Figure 1:**
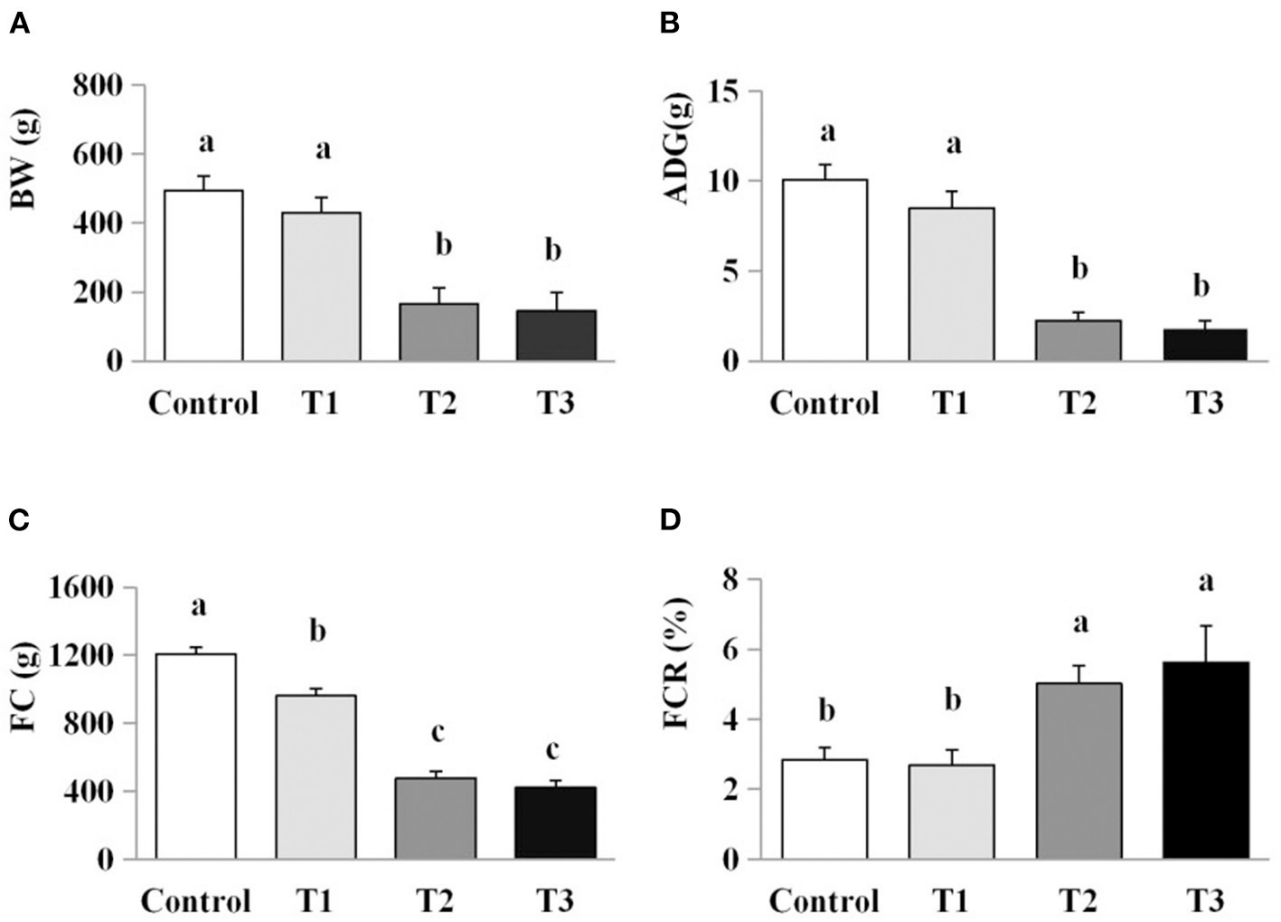
Effects of AFB_1_ on the growth performance of chicks. **(A)** BW, body weight. **(B)** ADG, average daily gain. **(C)** FC, feed consumption. **(D)** FCR, feed conversion ratio. Control: control group. T1: low doses of AFB_1_ group. T2: medium doses of AFB_1_ group. T3: high doses of AFB_1_ group. Data represent the means ± SD values of six replicate cages and were compared using one-way analysis of variance (ANOVA) followed by Duncan's multiple comparison tests. Different letters on the same row indicate significant differences (*P* < 0.05).

A significant reduction in FC and ADG were observed in the chicks exposed to the lowest dose of AFB_1_ (T1) compared with the control group (both *P* < 0.05). Further reductions in FC and ADG were observed in chicks given feed containing higher doses of AFB_1_ (T2 and T3) compared with the control and groups T1 (*P* < 0.05). Significantly higher FCR was observed in the T2 and T3 group than in the control and T1 groups (*P* < 0.05; Figure 1).

### Exposure to AFB_1_ dose-dependently affected oxidative stress

**Figure 2:**
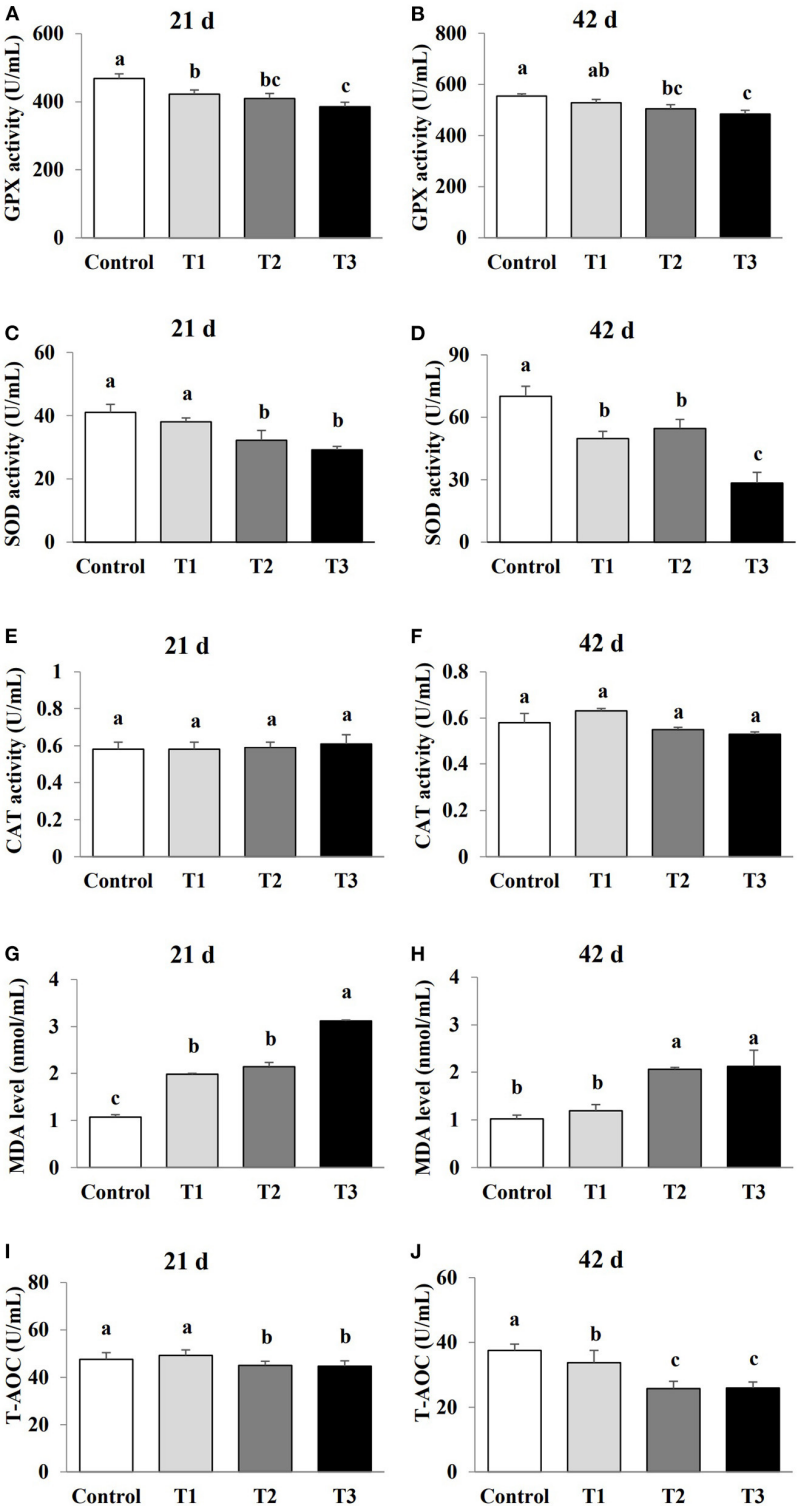
Effects of AFB_1_ on the antioxidant parameters in serum of chicks on day 21 and day 42. **(A)** GPX activity on day 21. **(B)** GPX activity on day 42. **(C)** SOD activity on day 21. **(D)** SOD activity on day 42. **(E)** CAT activity on day 21. **(F)** CAT activity on day 42. **(G)** MDA content on day 21. **(H)** MDA content on day 42. **(I)** T-AOC activity on day 21. **(J)** T-AOC activity on day 42. Control: control group. T1: low doses of AFB_1_group. T2: medium doses of AFB_1_ group. T3: high doses of AFB_1_ group. Data represent the means ± SD values of six replicate cages and were compared using one-way analysis of variance (ANOVA) followed by Duncan's multiple comparison tests. Different letters on the same row indicate significant differences (*P* < 0.05).

The effect of AFB_1_ exposure on oxidative stress was examined by comparing the levels of oxidative stress markers (GPX, SOD, CAT, MDA and T-AOC) in the sera of the control and AFB_1_-treated chicks. The GPX and SOD activities in the AFB_1_ treatment groups (T1, T2, and T3) were reduced in a dose-dependent manner at days 21 and 42 compared with the control (*P* < 0.05; Figure 2). A dose-dependent reduced in T-AOC levels was also observed in the AFB_1_ treatment groups (T1, T2, and T3) on day 42 compared with that of the control group (*P* < 0.05). In contrast, the MDA levels in the AFB_1_ treatment groups (T1, T2, and T3) increased dose-dependently relative to those in control group on days 21 and 42 (*P* < 0.05; Figure 2). CAT activity was unaffected by any AFB_1_ treatment.

### Exposure to AFB_1_ dose-dependently reduced serum immunoglobulin levels

**Figure 3:**
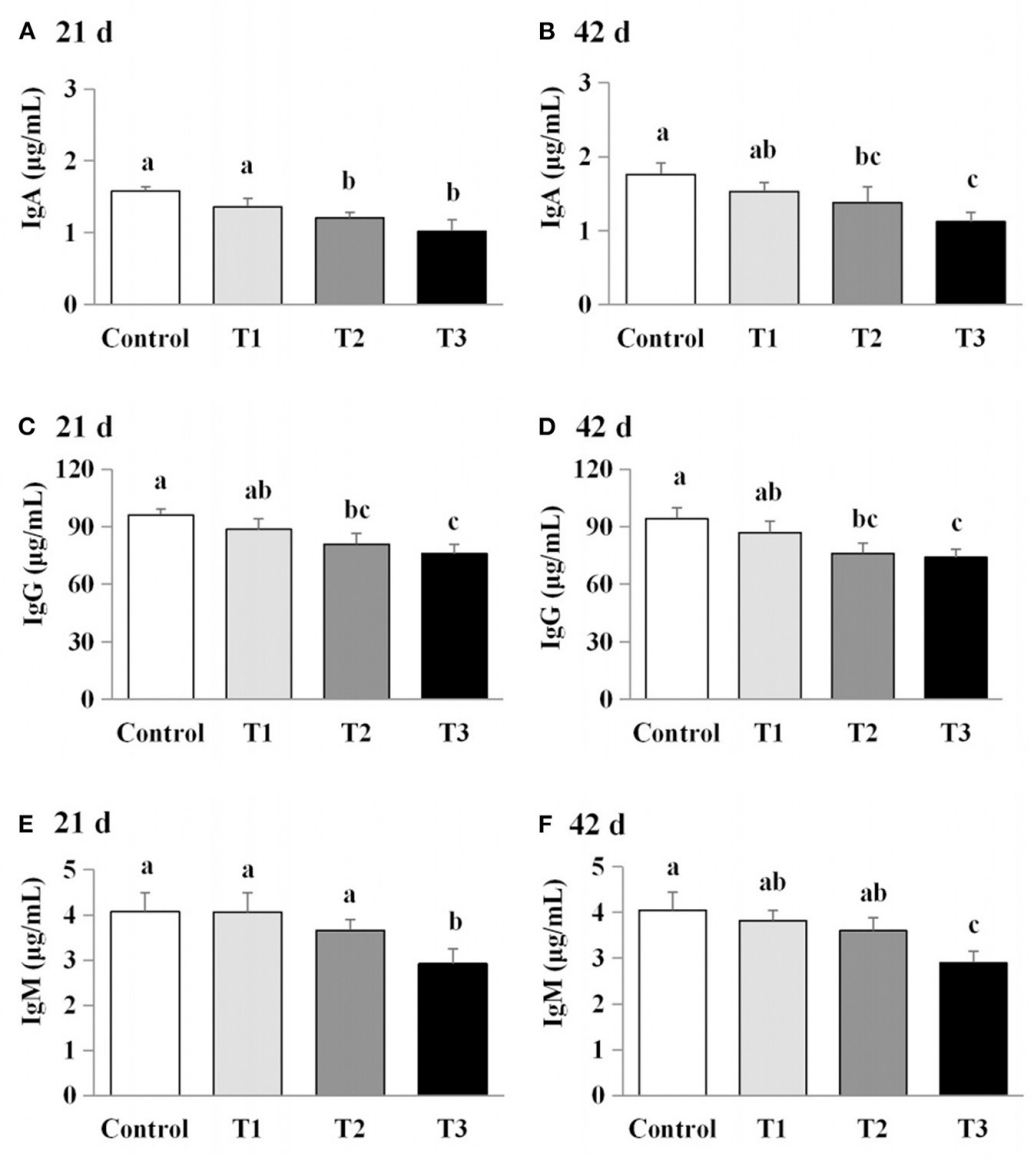
Effects of AFB_1_ on the immunoglobulin content in serum of chicks on day 21 and day 42 **(A)** IgA content on day 21. **(B)** IgA content on day 42. **(C)** IgG content on day 21. **(D)** IgG content on day 42. **(E)** IgM content on day 21. **(F)** IgM content on day 42. Control: control group. T1: low doses of AFB_1_group. T2: medium doses of AFB_1_ group. T3: high doses of AFB_1_ group. Data represent the means ± SD values of six replicate cages and were compared using one-way analysis of variance (ANOVA) followed by Duncan's multiple comparison tests. Different letters on the same row indicate significant differences (*P* < 0.05).

The effects of AFB_1_ exposure on immunoglobulin levels (IgA, IgG, and IgM) in the sera of the control and AFB_1_-treated chicks were determined. The IgA and IgG levels in the AFB_1_-treatment groups (T1, T2, and T3) were significantly reduced in a dose-dependent manner at days 21 and 42 compared with those in the control (*P* < 0.05; Figure 3). A significant dose-dependent reduction in IgM was observed in the AFB_1_-treated chicks on day 42 compared with the controls (*P* < 0.05; Figure 3).

### Exposure to AFB_1_ dose-dependently reduced serum cytokine levels

**Figure 4:**
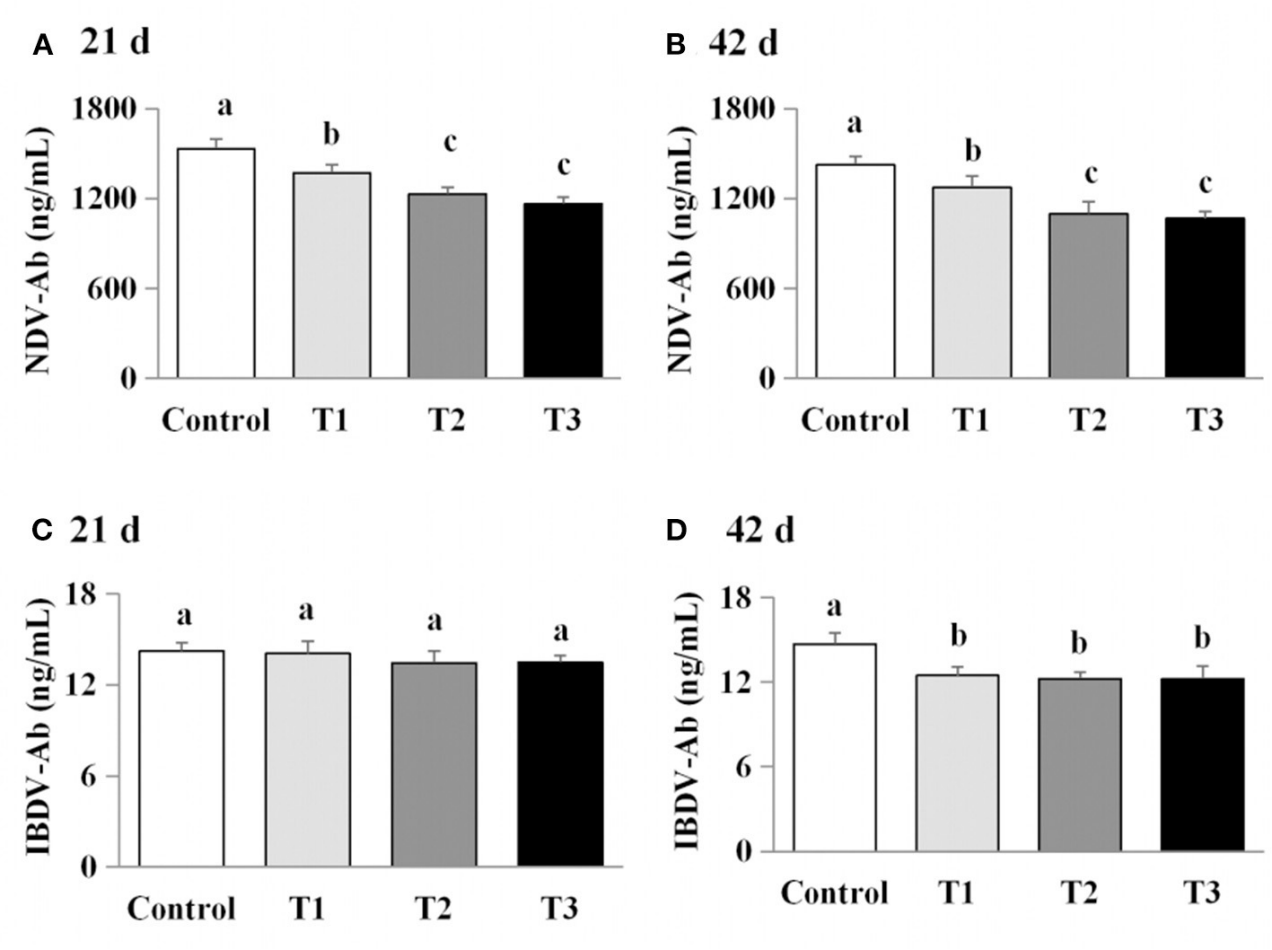
Effects of AFB_1_ on the antibody content in serum of chicks on day 21 and day 42. **(A)** IBDV-Ab content on day 21. **(B)** IBDV-Ab content on day 42. **(C)** NDV-Ab content on day 21. **(D)** NDV-Ab content on day 42. Control: control group. T1: low doses of AFB_1_group. T2: medium doses of AFB_1_ group. T3: high doses of AFB_1_ group. Data represent the means ± SD values of six replicate cages and were compared using one-way analysis of variance (ANOVA) followed by Duncan's multiple comparison tests. Different letters on the same row indicate significant differences (*P* < 0.05).

The effects of AFB_1_ exposure on cytokine levels (IL-2, IL-6, and IFN-γ) in the sera of the control and AFB_1_-treated chicks were determined. The IL-2, IL-6, and IFN-γ levels in the AFB_1_-treatment groups (T1, T2, and T3) were significantly and dose-dependently lower than those in the control group on days 21 and 42 (*P* < 0.05) (Figure 4).

### Exposure to AFB_1_ dose-dependently reduced serum antibody titers

**Figure 5:**
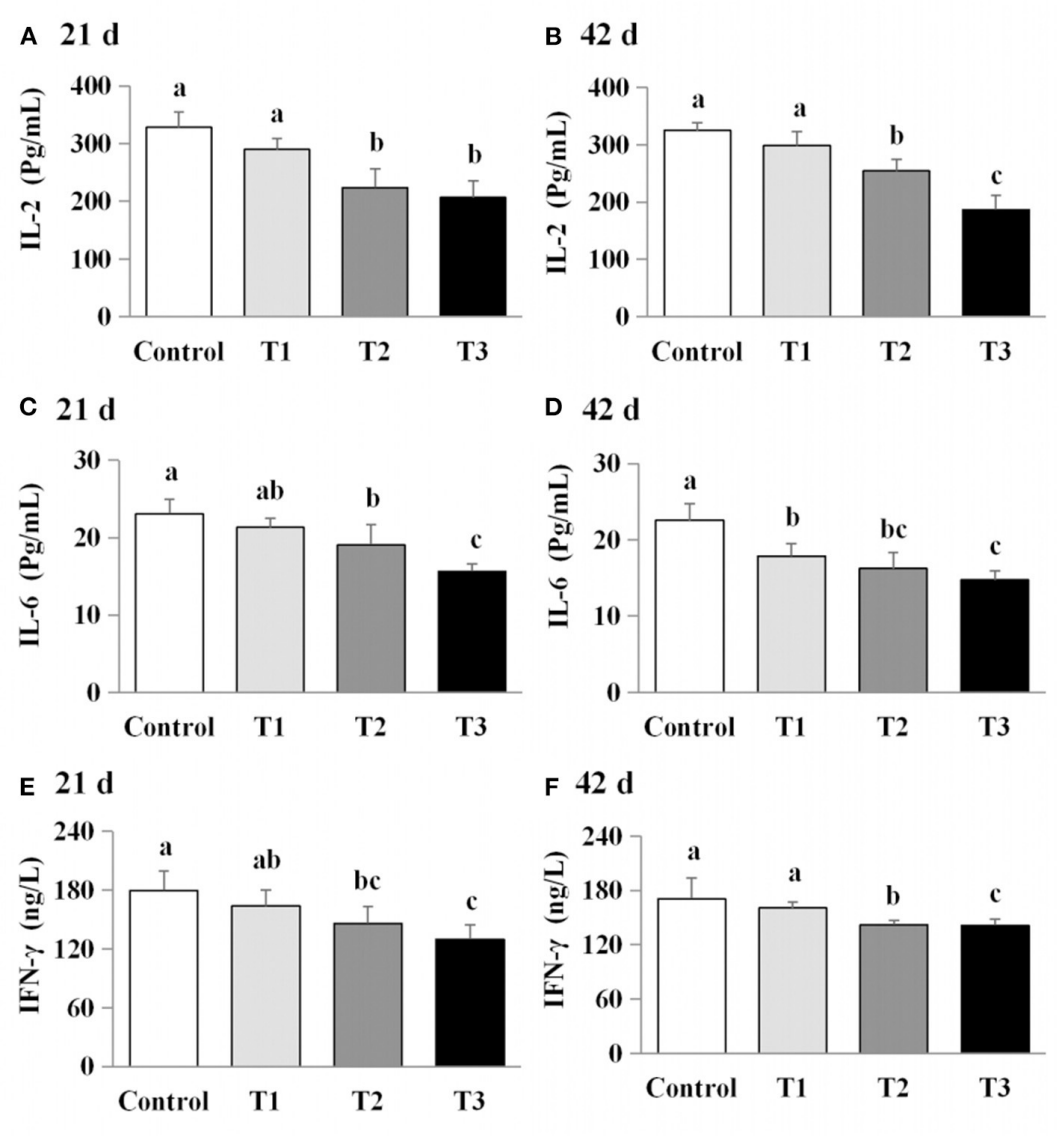
Effects of AFB1 on the immune factors content in serum of chicks on day 21 and day 42. **(A)** IL-2 content on day 21. **(B)** IL-2 content on day 42. **(C)** IL-6 content on day 21. **(D)** IL-6 content on day 42. **(E)** IFN-γ content on day 21. **(F)** IFN-γ content on day 42. Control: control group. T1: low doses of AFB_1_group. T2: medium doses of AFB_1_ group. T3: high doses of AFB_1_ group. Data represent the means ± SD values of six replicate cages and were compared using one-way analysis of variance (ANOVA) followed by Duncan's multiple comparison tests. Different letters on the same row indicate significant differences (*P* < 0.05).

We next examined the effects of AFB_1_ on the titers of IBDV-Ab and NDV-Ab. Compared with the control group, the AFB_1_-treated chicks (T1, T2, and T3) had a lower IBDV-Ab on day 21, and lower NDV-Ab and IBDV-Ab on days 42 (*P* < 0.05) (Figure 5). Furthermore, the NDV-Ab levels were lower on days 21 and 42 in the chicks exposed to the higher AFB_1_ concentrations (T2 and T3) than in the lowest-dose AFB_1_-treatment group (T1; *P* < 0.05; Figure 5).

### Exposure to AFB_1_ dose-dependently altered in the expression of antioxidant enzymes and pro-inflammatory cytokines genes

**Figure 6:**
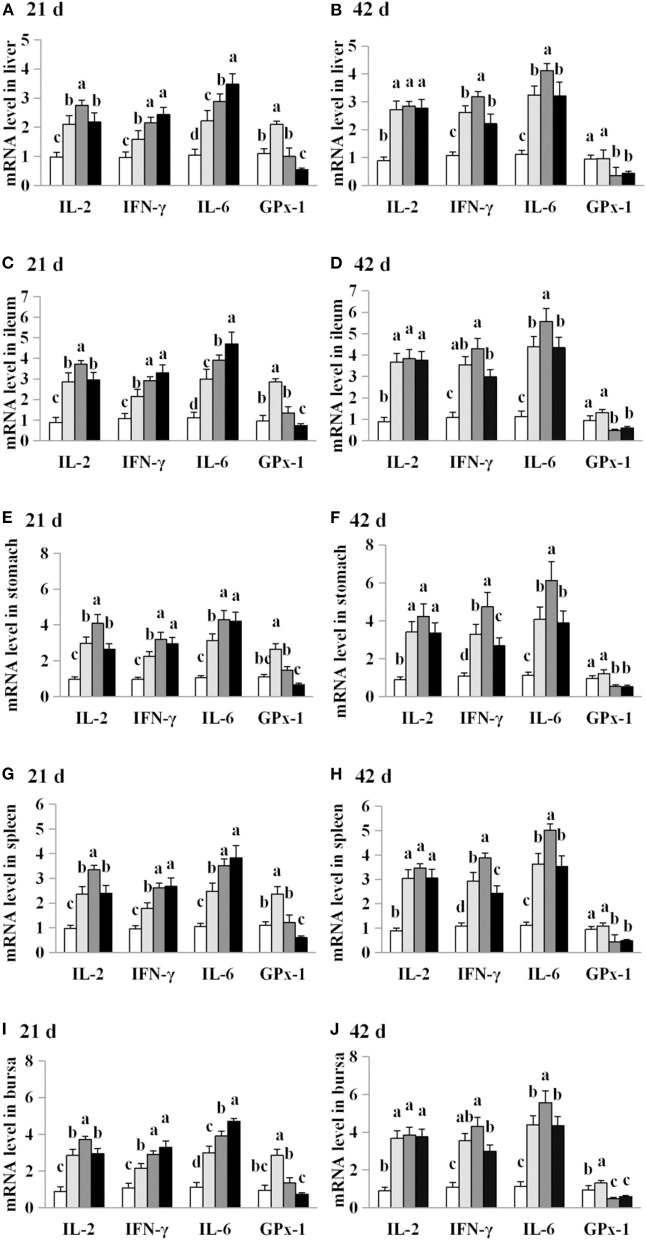
Effects of AFB1 on IL-2, IL-6, IFN-γ and GPx-1 mRNA levels in the liver, spleen, ileum, stomach and bursal of chicks on day 21 and day 42. **(A)** IL-2, IL-6, IFN-γ and GPx-1 mRNA levels in the liver on day 21. **(B)** IL-2, IL-6, IFN-γ and GPx-1 mRNA levels in the liver on day 42. **(C)** IL-2, IL-6, IFN-γ and GPx-1 mRNA levels in the spleen on day 21. **(D)** IL-2, IL-6, IFN-γ and GPx-1 mRNA levels in the spleen on day 42. **(E)** IL-2, IL-6, IFN-γ and GPx-1 mRNA levels in the ileum on day 21. **(F)** IL-2, IL-6, IFN-γ and GPx-1 mRNA levels in the ileum on day 42. **(G)** IL-2, IL-6, IFN-γ and GPx-1 mRNA levels in the stomach on day 21. **(H)** IL-2, IL-6, IFN-γ and GPx-1 mRNA levels in the stomach on day 42. **(I)** IL-2, IL-6, IFN-γ and GPx-1 mRNA levels in the bursal on day 21. **(J)** IL-2, IL-6, IFN-γ and GPx-1 mRNA levels in the bursal on day 42. Control: control group. T1: low doses of AFB_1_group. T2: medium doses of AFB_1_ group. T3: high doses of AFB_1_ group. Data represent the means ± SD values of six replicate cages and were compared using one-way analysis of variance (ANOVA) followed by Duncan's multiple comparison tests. Different letters on the same row indicate significant differences (*P* < 0.05).

The effects of AFB_1_ exposure on IL-2, IL-6, IFN-γ and GPx-1 mRNA levels in the livers, spleens, ileums and stomachs of control and AFB_1_-treated chicks were examined. IL-2, IL-6 and IFN-γ mRNA levels were significantly higher in all four organs of the AFB_1_-treated groups (T1, T2, and T3) than in those of the control group on days 21 and 42 (*P* < 0.05; Figure 6). On day 21, higher GPx-1 mRNA levels were observed in all four organs of the chicks exposed to the dose AFB_1_ treatment (T2) than in those of the control group. In contrast, reductions in the GPx-1 mRNA levels in all four organs were observed 21 days after treatment with the highest AFB_1_ dose (group T3) and in both the T2 and T3 groups at 42 days after treatment (*P* < 0.05) (Figure 6).

## Discussion

Growth performance is an important economical factor in all livestock industries, and is influenced by the effects of toxins on an animal's digestion and metabolism. In the present study, the concentrations of dietary AFB_1_ used in the diets examined here were based on the EUCR (14) and CHSF (15) guidelines for AFB_1_ concentrations in the feeds of young birds. The EUCR (14) AFB_1_ guideline recommends 5 μg/kg in dairy animal feeds and 10 μg/kg in feed for young animals, whereas the CHSF (15) AFB_1_ guideline recommends 10 μg/kg for feed for young animals. Here, we found that AFB_1_ supplementation led to a dose-dependent reduced in growth performance in chicks, measured as reductions in BW, ADG, and FC, and increase in FCR. These results are consistent with those reported in broiler chickens (6), where a dose-dependent effect particularly in feed intake was observed. Other meta-analyses have confirmed that the magnitude of these effects varied with the concentration of mycotoxins present in poultry and growing pigs (5, 16).

Numerous studies have reported that multiple mycotoxins, including AFB_1_, deoxynivalenol (DON), zearalenone (ZEN), and T-2 could induce oxidative stress (17, 18). Consistent with these studies, our findings indicated that AFB_1_ supplementation caused oxidative stress, leading to a-dose-dependent reductions in GPx-1 mRNA levels, T-AOC, and SOD and GPX activities, and dose-dependent increases in MDA levels, and demonstrated that the degree of oxidative stress was gradually enhanced as the level of AFB_1_ supplementation increased. As key enzymatic antioxidants, SOD and GPX, play important roles in eliminating reactive oxygen species (ROS) from cells (19–21). As the end product of lipid peroxidation, MDA is widely used as a late biomarker of oxidative stress and cellular damage (22). Our results suggested that the oxidative damage induced by AFB_1_ occurred mainly through an increase in lipid peroxidation and oxygen free radicals, in response to reduced SOD and GPX activities, and a reduction in GPx-1 mRNA levels. In contrast, Li et al. (23) reported that the oxidative damage induced by AFB_1_ occurred mainly through increased lipid peroxidation, but that it had no effect on antioxidant enzymes when administered to broiler chickens at a high concentration (74 μg/kg). Therefore, we hypothesize that chronic low doses of AFB_1_ may play an important role in inducing oxidative damage through the sensitive antioxidant enzymes in layer chicks.

The concentrations of IgA, IgG, and IgM and the titers of IBDV-Ab and NDV-Ab were previously found to decrease dose-dependently as the concentration of mycotoxins increased (5, 16). In the present study, our results confirmed that AFB_1_ supplementation significantly modulated the humoral immune response. Similarly, numerous studies have reported that multiple mycotoxins resulted in lower antibody titers (24, 25) and immunoglobulin concentrations (26) after vaccination. In contrast, other studies have demonstrated that many mycotoxins do not alter the concentrations of immunoglobulin subsets (24, 27, 28) and therefore failed to cause a significantly impair the specific humoral response after vaccination or sensitization (29–31). Therefore, different doses of AFB_1_ can cause distinct humoral immune responses, resulting in changes in immunoglobulin concentrations and antibody titers.

Cytokines are essential mediators of the inflammatory response and immune function. Macrophages, T cells and B cells are the central targets of multiple mycotoxins, which can be immunostimulatory or immunosuppressive (31, 32). Previous studies have shown that mycotoxin treatment can increase (25, 28, 33) or reduce inflammatory cytokine mRNA levels (22, 30, 34, 35). In the present study, AFB_1_ supplementation inhibited IL-2, IL-6, and IFN-γ protein production while promoting the mRNA expressions of these cytokines. Our findings are consistent with previous studies, which demonstrated that AFB_1_ treatment reduced the concentrations or mRNA levels of IL-2 or IL-4 but increased the gene expression of IL-1α, IL-6, IFN-γ, or TNF-α (7, 23). We consider that the effects of AFB_1_ on the inflammatory cytokines are dependent upon the dose administered, the duration of exposure, the susceptibility of each tissue, and the animal species examined, as well as other experimental conditions. Therefore, the increase in pro-inflammatory cytokine mRNA expression observed here may be attributable to the suppression of pro-inflammatory cytokines by AFB_1_ exposure.

In summary, AFB_1_ exposure, even at low levels, causes dose-dependent oxidative and inflammatory damage and immunosuppression, which reduced the growth performance of chicks.

## Data availability statement

The original contributions presented in the study are included in the article/supplementary material, further inquiries can be directed to the corresponding author.

## Ethics statement

All animal experiments were approved by Qingdao Agricultural University Animal Care and Use Committee (Qingdao, China) in accordance with Laboratory Animal-Guideline for ethical review of animal welfare (GB/T35892-2018, National Standards of the People's Republic of China).

## Author contributions

LH designed the study and discussed the results and wrote the paper. HQ contributed to the cell and animal experiment. AL contributed to interpretation of findings. JD contributed to the data analyses. LZ and GL contributed to reviewing of the manuscript. FC was the principal investigator and in charge of the whole trial. All authors read and approved the final version of the manuscript.

## Funding

This study was supported by Shandong Natural Science Foundation (ZR2021MC150), Open Project of Shandong Provincial Key Laboratory of Poultry Diseases Diagnosis and Immunology, and Shandong Modern Agricultural Technology and Industry System, China (SDAIT-11-07). Doctoral Fund project of Qingdao Agricultural University (663/1120017). These fund projects are publicly licensed and free of any potential commercial conflicts.

## Conflict of interest

The authors declare that the research was conducted in the absence of any commercial or financial relationships that could be construed as a potential conflict of interest.

## Publisher's note

All claims expressed in this article are solely those of the authors and do not necessarily represent those of their affiliated organizations, or those of the publisher, the editors and the reviewers. Any product that may be evaluated in this article, or claim that may be made by its manufacturer, is not guaranteed or endorsed by the publisher.
